# Broad-Scale Phosphoprotein Profiling of Beta Adrenergic Receptor (β-AR) Signaling Reveals Novel Phosphorylation and Dephosphorylation Events

**DOI:** 10.1371/journal.pone.0082164

**Published:** 2013-12-05

**Authors:** Andrzej J. Chruscinski, Harvir Singh, Steven M. Chan, Paul J. Utz

**Affiliations:** 1 Division of Cardiology and Heart Transplantation, Department of Medicine, Toronto General Hospital, Toronto, Ontario, Canada; 2 Developmental and Reproductive Biology, University of Hawaii, Honolulu, Hawaii, United States of America; 3 Division of Hematology, Department of Medicine, Stanford University, Stanford, California, United States of America; 4 Division of Immunology and Rheumatology, Department of Medicine, Stanford University, Stanford, California, United States of America; University of North Dakota, United States of America

## Abstract

β-adrenergic receptors (β-ARs) are model G-protein coupled receptors that mediate signal transduction in the sympathetic nervous system. Despite the widespread clinical use of agents that target β-ARs, the signaling pathways that operate downstream of β-AR stimulation have not yet been completely elucidated. Here, we utilized a lysate microarray approach to obtain a broad-scale perspective of phosphoprotein signaling downstream of β-AR. We monitored the time course of phosphorylation states of 54 proteins after β-AR activation mouse embryonic fibroblast (MEF) cells. In response to stimulation with the non-selective β-AR agonist isoproterenol, we observed previously described phosphorylation events such as ERK1/2(T202/Y204) and CREB(S133), but also novel phosphorylation events such as Cdc2(Y15) and Pyk2(Y402). All of these events were mediated through cAMP and PKA as they were reproduced by stimulation with the adenylyl cyclase activator forskolin and were blocked by treatment with H89, a PKA inhibitor. In addition, we also observed a number of novel isoproterenol-induced protein dephosphorylation events in target substrates of the PI3K/AKT pathway: GSK3β(S9), 4E-BP1(S65), and p70s6k(T389). These dephosphorylations were dependent on cAMP, but were independent of PKA and correlated with reduced PI3K/AKT activity. Isoproterenol stimulation also led to a cAMP-dependent dephosphorylation of PP1α(T320), a modification known to correlate with enhanced activity of this phosphatase. Dephosphorylation of PP1α coincided with the secondary decline in phosphorylation of some PKA-phosphorylated substrates, suggesting that PP1α may act in a feedback loop to return these phosphorylations to baseline. In summary, lysate microarrays are a powerful tool to profile phosphoprotein signaling and have provided a broad-scale perspective of how β-AR signaling can regulate key pathways involved in cell growth and metabolism.

## Introduction

 Beta-adrenergic receptors (β-AR) are G-protein coupled receptors that mediate the effects of the catecholamines, epinephrine and norepinephrine, in the sympathetic nervous system. Three β-AR subtypes have been identified (β1-AR, β2-AR, and β3-AR) [1–3]. These receptors are expressed throughout the target organs of the sympathetic nervous system including the heart, skeletal muscle, smooth muscle cells in the bronchi and digestive tract, and adipose tissue [1–3]. β-AR agonists are currently used as bronchodilators, tocolytic agents and chronotropic/inotropic agents, whereas β-AR antagonists or “β blockers” have revolutionized the treatment of a number of cardiovascular disorders including angina, hypertension, and heart failure [4,5]. More recently, it has been appreciated that drugs that target β-AR signaling can also modulate the growth and survival of various cell types including certain tumors [6–8]. Thus, unraveling the complex signaling mechanisms that occur downstream of β-ARs has the potential to impact the treatment of a number of diseases that currently burden our population.

It is known that upon binding agonist ligands, all three types of β-ARs couple to the stimulatory G-protein (Gs), resulting in the activation of adenylyl cyclase and generation of the second messenger cAMP [9]. cAMP then activates protein kinase A (PKA), which can phosphorylate a variety of target proteins [10]. Over the past several years, there has been an appreciation that there are effects of cAMP that are independent of PKA. This has led to the discovery of exchange proteins activated by cAMP (Epac) [11]. Using techniques of molecular cloning, two Epac subtypes have been identified (Epac1 and Epac2), which are both capable of activating the small G proteins Rap1 and Rap2 [12] and modulating a wide variety of cellular functions [13,14].

Whereas β1-ARs couple only to the stimulatory G-protein (Gs), β2-ARs have the ability to couple to both Gs and the inhibitory G-protein (Gi) [15]. Coupling to Gi has been shown in some studies to result from phosphorylation of the third intracellular loop of the β2-AR by PKA [16]. The unique ability of β2-AR to couple to Gi-dependent signaling pathways may account for some of the differences in biological effects of β1-AR and β2-AR agonists [7]. Clarity is lacking as to the exact nature of Gi-dependent signaling events. For example, some investigators have shown that β2-AR stimulation through Gi leads to phosphorylation of ERK1/2 [16], while others have shown that this signaling event is independent of Gi coupling and rather occurs through PKA activation [17]. Certainly, such findings highlight the need for a more complete characterization of signaling pathways downstream of β-AR stimulation.

In order to gain a more broad and unbiased perspective of β-AR signaling and to understand how the different mechanisms contribute to signaling, we utilized a lysate microarray approach to profile protein phosphorylation events downstream of β-AR in an immortalized mouse embryonic fibroblast (MEF) cell line. After stimulating MEFs with various β-AR agonists, lysates were harvested at different time-points and were used to construct lysate microarrays, which were probed with a panel of 54 well-characterized phospho-specific antibodies. Because a large number cell lysates were spotted onto the slides, many different stimulatory conditions could be assessed simultaneously, thus providing a broad-scale view of the kinetics of the β-AR signaling pathway. 

Using this approach, we identified a number of novel tyrosine phosphorylation events downstream of β-AR. We also identified novel dephosphorylation events downstream of β-AR and cAMP that were independent of PKA and Epac, but correlated with reduced AKT activity. Together, these studies shed light on the diverse and important cellular functions mediated by β-AR activation. Furthermore, they highlight the utility of lysate microarray technique to provide broad overviews of signaling and to uncover novel signaling events.

## Materials and Methods

### Cell Culture and Reagents

A MEF cell line [18] was maintained in DMEM (Life Technologies, Grand Island, NY, USA) containing 10% fetal calf serum (Life Technologies) and 1% penicillin-streptomycin (Life Technologies) in a 37°C incubator at 5% CO_2_. The following inhibitors and stimuli were purchased from Sigma-Aldrich (St. Louis, MO, USA) unless otherwise specified: isoproterenol, forskolin, 8-pCPT-2'-O-Me-cAMP (Biolog, Bremen, Germany), H89, LY294002 (Millipore, Billerica, MA, USA), wortmannin, okadaic acid, CGP 20712A, ICI 118,551, pertussis toxin, prazosin, epinephrine, and norepinephrine. All antibodies used in this study were purchased from Cell Signaling Technologies (Danvers, MA, USA) unless otherwise specified. A complete list of antibodies used in this study is available online (http://utzlab.stanford.edu/protocols/). 

### Stimulation Experiments and Lysate Preparation

For stimulation experiments, cells were seeded into 6-well plates. After the cells were confluent, they were serum starved overnight and were stimulated for various times. Following stimulations, cells were washed with ice-cold PBS and then lysed in buffer containing 50 mM Tris pH 6.8, 2% SDS, 5% glycerol, 1% 2-mercaptoethanol, 2.5 mM EDTA, 1.5x Halt phosphatase inhibitor cocktail (Thermo Fisher Scientific, Waltham, MA, USA), and 1x complete protease inhibitor cocktail (Roche, Basal, Switzerland). Lysate samples were immediately snap-frozen in a dry ice/ethanol bath and stored at -80°C. Prior to printing, lysates were boiled at 100°C for 10 minutes and were briefly centrifuged. Protein levels of all lysates were quantified using the Quant-It Protein Assay Kit (Life Technologies) and diluted with additional lysis buffer to equalize protein concentrations. Samples were loaded into wells of a 384-well plate in preparation for printing.

### Lysate Microarray Printing and Processing

Lysate microarrays were printed and processed as previously described [19]. Briefly, lysates were spotted in triplicate onto nitrocellulose-coated FAST slides (GE Healthcare, Little Chalfont, UK) using a VersArray ChipWriter Compact Arrayer (Bio-Rad Laboratories, Hercules, CA, USA) with solid pins (Arrayit Corporation, Sunnyvale, CA, USA). Lysates from individual time course experiments were printed with the same solid pin. After the lysate microarrays were dry, they were placed in FAST frames (GE Healthcare, Little Chalfont, UK) and were blocked in a 3% casein solution (Bio-Rad Laboratories, Hercules, CA, USA) for 3-4 hours at room temperature. Slides were probed with primary antibodies overnight at 4°C in dilution buffer (PBS, 20% FCS, 0.1% Tween). Following extensive washing, the slides were incubated at room temperature with anti-rabbit IgG HRP-conjugated secondary antibody (Jackson ImmunoResearch, West Grove, PA, USA) in dilution buffer for 1 hour. The signal was amplified by incubating with 1x Bio-Rad Amplification Reagent supplied in the Amplified Opti-4CN Substrate Kit (Bio-Rad Laboratories) for 10 minutes at room temperature. Following amplification, the slides were probed with streptavidin Alexa Fluor-647 conjugate (Life Technologies) for 1 hour. Slides were dried with an aspirator and scanned using a GenePix 4000B microarray scanner (Molecular Devices, Sunnyvale, CA, USA).

### Microarray Data Analysis

Scanned slides were analyzed using GenePix Pro Version 6.0 software (Molecular Devices). The median fluorescence intensity of each feature minus background (*MFI-B*) was determined and averaged to obtain a fluorescence value for each lysate representing a single time point from a time course experiment. The values for each lysate were normalized to the *MFI-B* values of the respective vehicle-treated control spots providing a fold change in phosphorylation state. These data were imported into TIGR Multiple Experiment Viewer software (TMEV) [20] to create heat map representations and to perform hierarchical clustering, and significance analysis as described. Time-course (two class unpaired) Significance Analysis of Microarrays (SAM) [21] was applied to the data to identify pathways significantly (q<0.05) altered by various inhibitors. 

### Western Blotting

Lysates were loaded into wells of 12% Bis-Tris Mini-Gels (Life Technologies), separated by polyacrylamide gel electrophoresis, and transferred onto a nitrocellulose membrane. Membranes were blocked in TBS, 0.1% Tween, and 5% milk for 1 hour at room temperature and incubated with primary antibody in TBS, 0.1% Tween, and 5% BSA overnight at 4°C. Membranes were probed with a donkey anti-rabbit HRP conjugated secondary antibody in blocking buffer for 1 hour at room temperature. The signal was visualized using a chemiluminescent ECL Western Blotting Detection kit (GE Healthcare). 

### Rap1 Pull-Down Assay

MEFs were serum starved overnight prior to stimulation with forskolin or 8-pCPT-2'-*O*-Me-cAMP (BioLog). Cells were stimulated for indicated time points and were lysed with ice-cold Rap1 activation lysis buffer containing 50 mM Tris·HCl (pH 7.4), 500 mM NaCl, 2.5 mM MgCl_2_, 10% glycerol, 1% Nonidet P-40 50mM, and 1x complete protease inhibitor cocktail (Roche). Cell lysates were briefly centrifuged and incubated for 45 minutes at 4°C with recombinant human Ral GDS-Rap Binding Domain (RBD) bound to glutathione-agarose beads (Millipore). Agarose beads were washed three times with Rap1 lysis buffer and were resuspended in 2x Laemmli reducing sample buffer. Samples were separated by SDS-PAGE, transferred onto nitrocellulose, and incubated with anti-Rap1 polyclonal antibody (Millipore) according to the western protocol described above.

### In Vitro AKT Kinase Assay

AKT kinase assays were performed using a nonradioactive kit from Cell Signaling Technology. Briefly, MEFs were grown in 10 cm dishes and treated with either vehicle control, LY294002 (10 μM), wortmannin (100 nM), or forskolin (50 μM) for 30 minutes. Following treatment, the cells were lysed in 1x cell lysis buffer supplemented with PMSF and were sonicated. The cell lysate was then centrifuged and the supernatant was stored at -80°C. After thawing, the cell lysates were incubated with an immobilized phospho-AKT (S473) (Cell Signaling Technology) antibody overnight at 4°C. The immunoprecipiates were then washed twice with lysis buffer (Cell Signaling Technology) and twice with kinase buffer (Cell Signaling Technology). The in vitro kinase reaction was carried out in 50 μl of kinase buffer containing immunoprecipitated AKT, 200 μM ATP, and GSK-3 fusion protein that served as the substrate. After an incubation of 30 min at 30°C, the reaction was stopped by the addition of SDS sample buffer. Samples were boiled for 5 min, were separated by SDS-PAGE, and were transferred to nitrocellulose membranes. The level of phosphorylated GSK-3 fusion protein was detected using an anti-phospho-GSK3α/β (S9/21) polyclonal antibody (1:1000 dilution; Cell Signaling Technology). The binding of primary antibody was visualized using an HRP-conjugated anti-biotin antibody and ECL reagent supplied with the kit. Western blot intensities were quantified by densitometry using Quantity One software (Bio-Rad Laboratories) and a GS-800 Calibrated Densitometer (Bio-Rad Laboratories). Statistical significance was assessed using a one-way ANOVA test followed by a Tukey’s post-hoc analysis for group comparisons.

### Radioligand Binding Assay

MEFs were grown to confluence, collected in lysis buffer (10 mM Tris-HCl, 1 mM EDTA, pH 7.4) with a cell scraper, and then subjected to dounce homogenization. Following a low speed centrifugation (500 x g) to remove cellular debris, membranes were pelleted by a high speed centrifugation (13,000 x g). Membranes were then resuspended in binding buffer (75 mM Tris-HCl, 12.5 mM MgCl_2_, 1 mM EDTA, pH 7.4). Binding reactions were carried out by incubating 60 μg of membranes with 10 nM [^3^H]dihydroalprenolol hydrochloride (PerkinElmer, Waltham, MA, USA) and different concentrations of ICI 118,551 (Sigma-Aldrich). After a two hour incubation at room-temperature, the binding reactions were terminated by rapid filtration over glass fiber filters (Millipore). Radioactivity in the filters was then quantified using a liquid scintillation counter. Non-specific binding was determined in the presence of 1 μM alprenolol (Sigma-Aldrich). Binding data were analyzed with GraphPad Prism software (GraphPad Software Inc., La Jolla, CA, USA).

## Results

**Figure 1:**
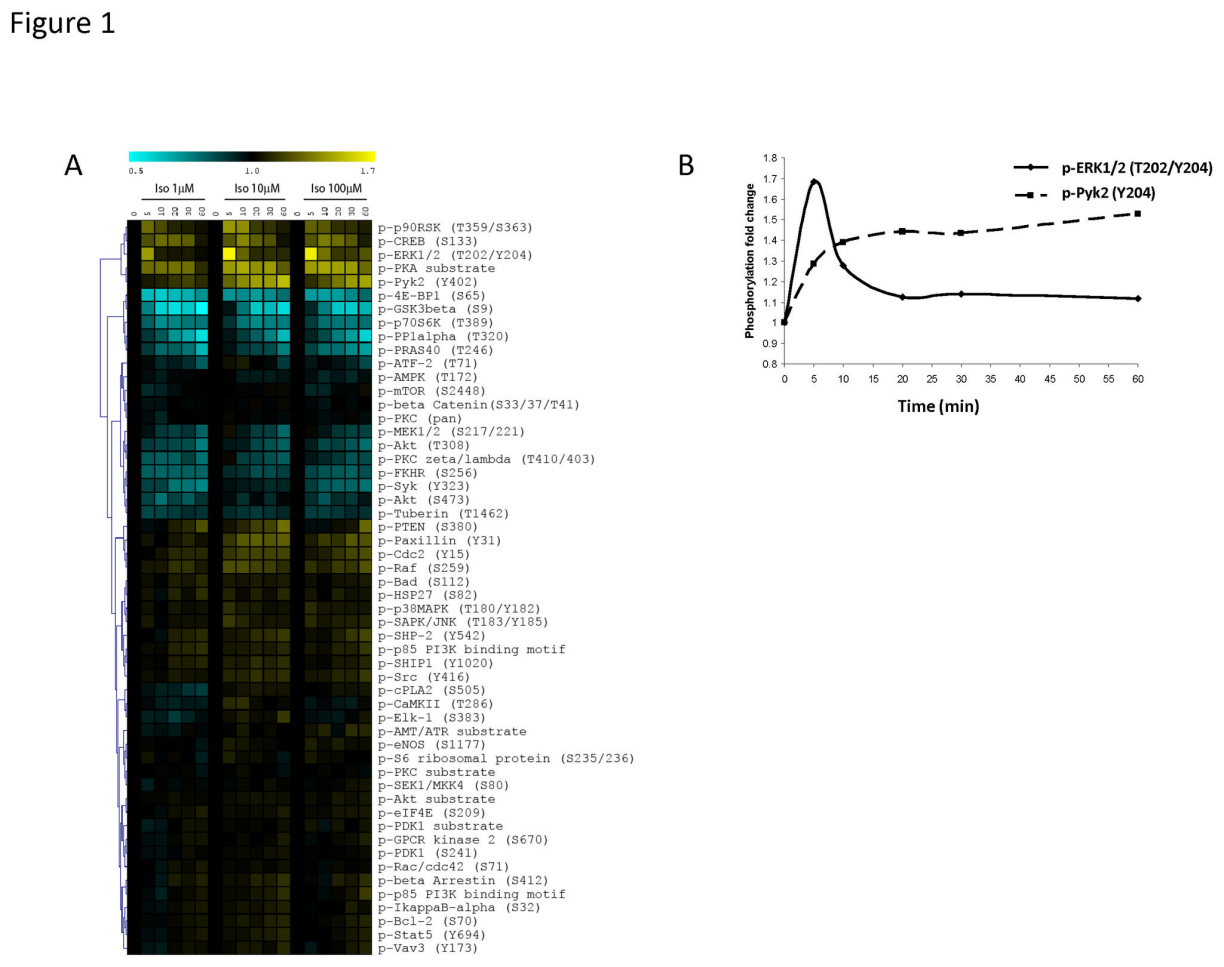
Phosphoprotein signaling in MEFs after stimulation with isoproterenol. **A**, Heat map representation of phosphoprotein changes over time. MEFs were grown to confluence and then were stimulated with different concentrations of isoproterenol (Iso) (1 μM, 10 μM, or 100 μM) for various times (0, 5, 10, 20, 30, or 60 minutes). Lysate microarrays were used to screen the MEF lysates against a panel of phospho-specific antibodies. Using data from the lysate microarrays, a heat map was constructed that revealed distinct clusters of phosphorylation (yellow) and dephosphorylation (blue) events after isoproterenol stimulation. The color scale shows fold change as compared with unstimulated MEFs. Data are representative of two independent experiments. **B**, Depiction of ERK1/2(T202/Y204) and Pyk2(Y402) phosphorylation kinetics over a 1 hour time course with isoproterenol (10 μM).

In order to characterize the protein phosphorylation events that operate downstream of β-ARs, we stimulated MEFs with different doses of the non-selective β-AR agonist isoproterenol or the endogenous agonists epinephrine and norepinephrine and then harvested lysates from these cells after various times (from 5 to 60 minutes). MEFs were used as a model system because they express a number of β-ARs and because β-AR signaling has been previously studied in these cells [22]. MEFs also provide a good platform for future studies because it is easy to grow these cells from various knockout mice to further dissect signaling mechanisms. Based on ligand binding studies, we confirmed that the majority (74.7%) of β-AR in MEFs are the β2-AR (Figure S1 in File S1). This result is similar to a prior study that showed that the β2-AR is the major β-AR subtype coupled to cAMP accumulation in primary MEFs [23]. Following stimulations, MEF cell lysates were printed onto microarrays, which were then probed individually with a panel of phospho-specific antibodies to identify phosphoprotein changes. Figure 1A, Figure S2 in File S1, and Figure S3 in File S1 show heat maps that were generated from these lysate microarray data. 

We detected many protein phosphorylation and dephosphorylation events after isoproterenol stimulation (Figure 1A). The pattern of these changes with isoproterenol was reproduced by stimulation with the endogenous catecholamines, epinephrine and norepinephrine (Figure S2 in File S1and Figure S3 in File S1). In addition to known phosphorylation events such as the phosphorylation of threonine 202 and tyrosine 204 (T202/Y204) on extracellular signal-regulated kinase 1/2 (ERK1/2) and the phosphorylation of serine 133 (S133) on cAMP response element binding protein (CREB), novel tyrosine phosphorylation events were detected after isoproterenol stimulation such as Y15 on cell division cycle 2 (Cdc2) and Y402 on proline-rich tyrosine kinase 2 (Pyk2). We also observed that the kinetics of these phosphorylations varied in a molecule-specific way. For example, we detected a rapid phosphorylation of ERK1/2(T202/Y204) signaling kinase within 5 minutes of stimulation followed by dephosphorylation of the protein back to baseline within 20 minutes. On the other hand, the phosphorylation of Cdc2(Y15) and Pyk2(Y402) occurred on a slower timescale (Figure 1B). In addition, lysate microarrays also revealed a number of dephosphorylation events in MEFs downstream of β-AR stimulation that have not yet been described: S9 on glycogen synthase kinase 3β (GSK3β), S65 on eukaryotic translation initiation factor 4E binding protein (4E-BP1), T389 on p70 ribosomal S6 kinase (p70S6K), and T320 on protein phosphatase 1α (PP1α). In order to validate these lysate microarray results, we performed traditional western blot analysis of phosphoprotein expression in these same MEF lysates and observed similar changes in the patterns of phosphorylation (Figure S4 in File S1). 

Based on prior studies that showed that the β2-AR is able to couple to the inhibitory G-protein Gi and phosphorylate ERK1/2 [15,16], we also conducted isoproterenol stimulations in the presence and absence of the Gi inhibitor, pertussis toxin. Based on results obtained using the SAM algorithm, we detected no significant differences between isoproterenol stimulations with and without pertussis toxin, indicating that the majority of signaling events that we observed in response to isoproterenol, including ERK1/2(T202/Y204), were independent of Gi and likely were dependent on Gs (Figure S5 in File S1). 

**Figure 2:**
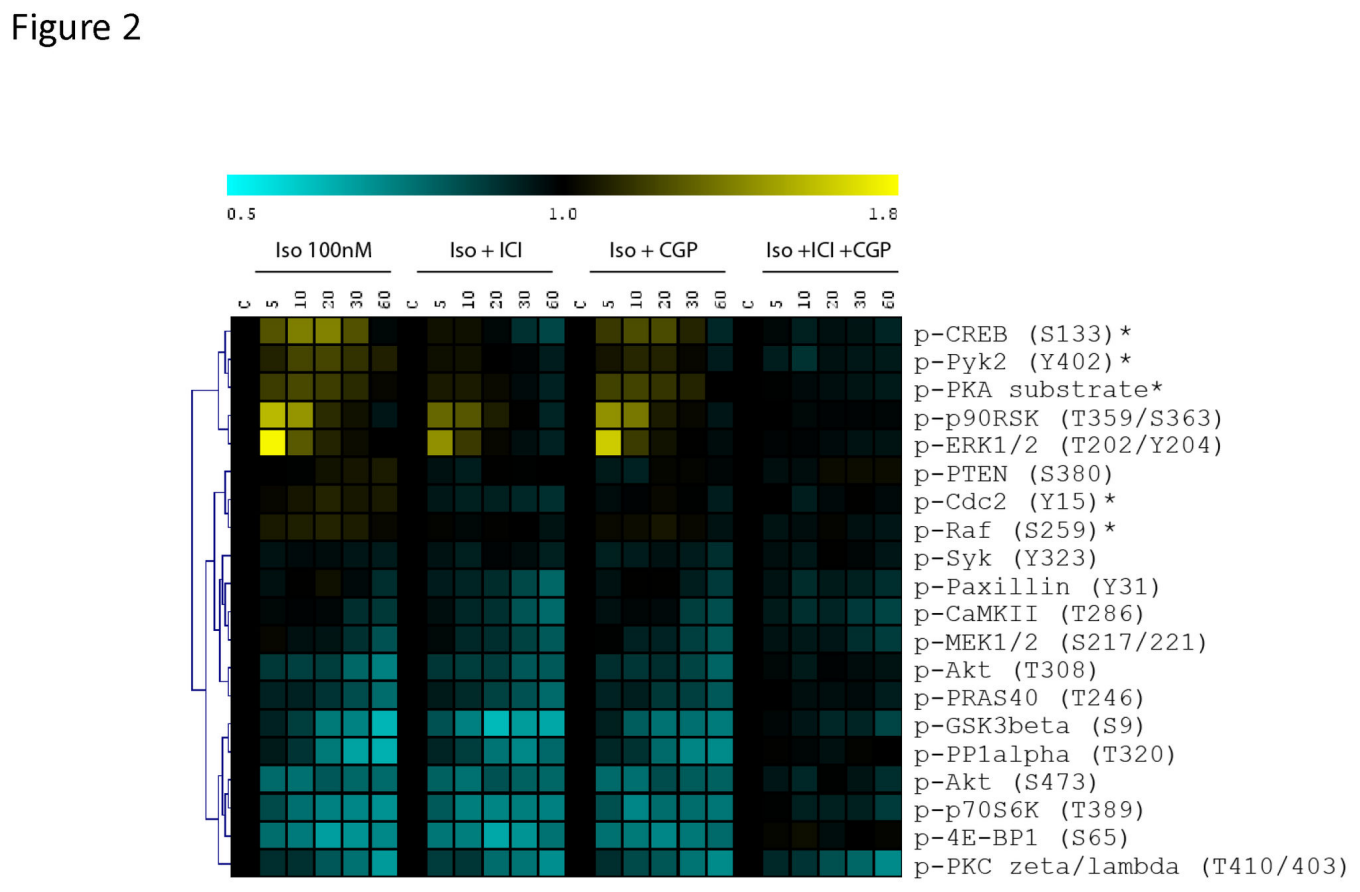
Isoproterenol induced phosphoprotein signaling in the presence of selective β-AR antagonists. MEFs were pretreated with the β1-AR antagonist CGP 20712A (CGP) and β2-AR antagonist ICI 118,551 (ICI) prior to stimulation with isoproterenol. CGP and ICI were used at final concentrations of 10 μM. MEFs were then stimulated with isoproterenol (Iso) (100 nM) for various times (0, 5, 10, 20, 30, or 60 minutes). The heat map shows phosphorylation (yellow) and dephosphorylation (blue) events after isoproterenol stimulation. The color scale shows fold change as compared with unstimulated MEFs. Data are means of three independent stimulation experiments. * denotes pathways with reduced phosphorylation in the presence of ICI 118,551 as determined using the SAM algorithm (q value < 0.05).

Next, we used specific antagonists of β1-AR (CGP 20712A) and β2-AR (ICI 118,551) to distinguish the role of these β-AR subtypes in mediating phospho-protein signaling downstream of isoproterenol stimulation in MEFs. For these experiments we focused on the signaling pathways that showed the greatest changes in phosphorylation status in our primary screen. We found that pre-treatment with β2-AR antagonist, but not the β1-AR antagonist significantly blunted the isoproterenol-induced phosphorylation of Cdc2(Y15), Pyk2(Y402), CREB(S133), Raf(S259), and PKA substrate (q values < 0.05), confirming that these phosphorylation events are coupled more to the β2-AR signaling pathway (Figure 2). Interestingly, we found that neither of these antagonists when used in isolation had an effect on the dephosphorylation of the signaling molecules. However, combined β1-AR and β2-AR inhibition significantly blocked the protein dephosphorylations, suggesting that these events are likely regulated by a shared signaling intermediate, such as a common pool of cAMP. The finding that the β1-AR antagonist countered the residual phosphorylation changes induced by isoproterenol further suggests that β1-AR may be the next dominant subtype in these cells. The only exception to this was PKCζ/λ(T410/403), which was still dephosphorylated after the addition of isoproterenol even in the presence of combined β1-AR and β2-AR blockade. Although not examined in this study, we speculate that the dephosphorylation of PKCζ/λ(T410/403) may potentially result from activation of β3-AR in MEFs. 

**Figure 3:**
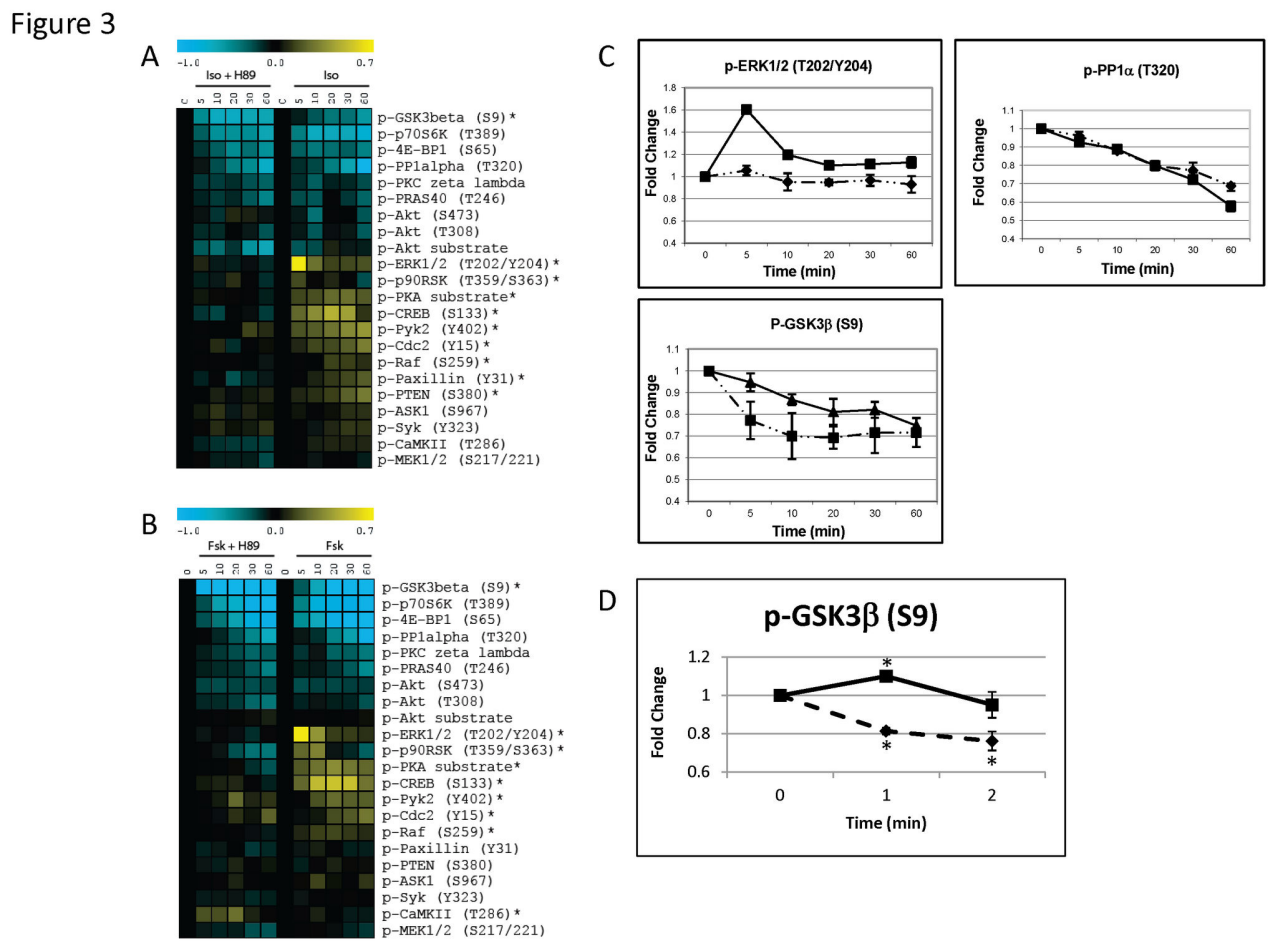
Isoproterenol- and forskolin-induced phosphoprotein signaling in the presence of the PKA inhibitor H89. **A**, MEFs were pretreated with H89 (10 μM) for 1 hour and then were stimulated with isoproterenol (Iso) (10 μM) for various times (0, 5, 10, 20, 30, or 60 minutes). The heat map shows phosphorylation (yellow) and dephosphorylation (blue) events after isoproterenol stimulation. The color scale shows fold change as compared with unstimulated MEFs. Data are means of three independent stimulation experiments. * denotes pathways that are significantly altered by pretreatment with H89 as assessed using the SAM algorithm. A two-class unpaired time course analysis was performed comparing signed-areas under the curve for each pathway. **B**, MEFs were pretreated with H89 as in (A) but were stimulated with forskolin (50 μM). Data are means of three independent stimulation experiments. * denotes pathways that are significantly altered by pretreatment with H89 as assessed using the SAM algorithm. **C**, The phosphorylation kinetics of ERK1/2(T202/Y204), PP1α(T320), GSK3β(S9) in response to isoproterenol (10 μM) stimulation in the presence (dashed line) or absence (solid line) of H89. The phosphorylation of ERK1/2(T202/Y204) is blocked by H89, dephosphorylation of PP1α(T320) is unchanged by H89, and dephosphorylation of GSK3β(S9) is enhanced by H89. Graphs show means ± SEM for three independent experiments. Values in the graph represent fold change as compared with unstimulated MEFs. D, Rapid phosphorylation kinetics of GSK3β(S9) by isoproterenol (10 μM) in the presence (dashed line) or absence (solid line) of H89. The graphs show means ± SEM of three independent experiments. * indicates a significant difference (P<0.05) from baseline phosphorylation level as determined using a Student’s T-Test.

To determine whether the isoproterenol-induced phosphorylation and dephosphorylation events are dependent on protein kinase A (PKA), we next pretreated MEFs with the PKA inhibitor H89 prior to conducting these stimulations. As seen in Figure 3A, the majority of the isoproterenol induced phosphorylation events were inhibited by pretreatment with H89 (see * in Figure 3A). On the other hand, the dephosphorylation events were largely unaffected by pretreatment with the H89, with the exception of GSK3β(S9) which occurred more rapidly under these conditions (Figures 3A and 3C). We speculate that the more rapid dephosphorylation of GSK3β(S9) in the presence of H89 is due to inhibition of the competing early PKA-mediated phosphorylation event because H89 inhibited a modest, yet rapid (1 minute) phosphorylation event on GSK3β(S9) (Figure 3D). 

To investigate the cAMP-dependence of these phosphoprotein changes, we also stimulated MEFs with forskolin, a direct activator of adenylyl cyclase. As can be seen in Figure 3B, forskolin produced similar phosphorylation and dephosphorylation signaling changes as compared with isoproterenol, confirming that all of these events are cAMP-dependent. As with the isoproterenol stimulations, the forskolin-induced phosphorylation events were inhibited by H89, whereas the forskolin-induced dephosphorylation events were largely unaffected by H89 with the exception of GSK3β(S9) dephosphorylation, which again was found to occur more extensively with H89 treatment (Figure 3B). 

Our studies using H89 and forskolin suggested that the dephosphorylation events induced by isoproterenol were dependent on cAMP, but were independent of PKA. We then reasoned that the cAMP sensor Epac may be a potential effector of these changes. To investigate Epac involvement, we treated MEFs with the Epac agonist 8-pCPT-2'-O-Me-cAMP (100 μM to 1 mM) to see if we could recapitulate the isoproterenol-induced dephosphorylation events. Surprisingly, stimulation of MEFs with this agent had no effect on either phosphorylation or dephosphorylation of signaling molecules (data not shown). In order to verify that 8-pCPT-2'-O-Me-cAMP was active in MEF cells, Rap GAP assays were also performed. These experiments confirmed that 8-pCPT-2'-O-Me-cAMP activated Rap1 similarly to forskolin in MEFs (Figure S6 in File S1). These results suggest that the cAMP-dependent dephosphorylations that we observed did not occur through Epac activation.

**Figure 4:**
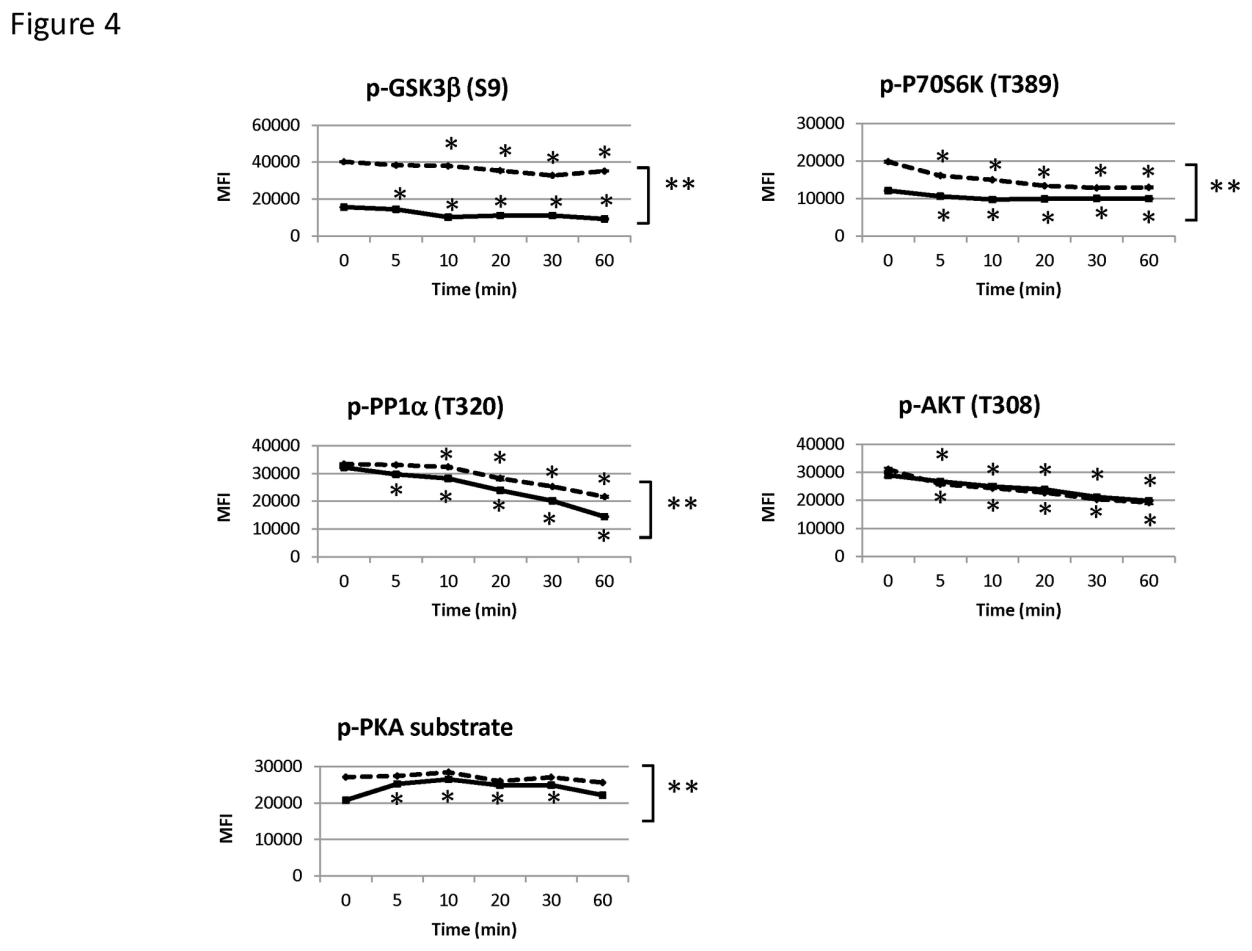
Isoproterenol-induced dephosphorylation events in the presence of okadaic acid. MEFs were pretreated with okadaic acid (1 μM) for 30 minutes and then were stimulated with isoproterenol (10 μM) for various times (0, 5, 10, 20, 30, or 60 minutes). Graphs show dephosphorylation of GSK3β(S9), P70S6K(T389), PP1α(T320), and AKT(T308) after the addition of isoproterenol in the presence (dashed line) or absence (solid line) of okadaic acid. The basal levels of phosphorylation of GSK3β(S9), P70S6K(T389), and PKA substrate were increased by okadaic acid at the unstimulated (0 minute) time point. Values in graphs are median ± S.D fluorescence intensity (MFI). of lysate microarray features. ** denotes a significant difference (P<0.05) from okadaic acid treatment as determined using a one-way ANOVA with repeated measures. * denotes significant difference (P<0.05) from the baseline time point as determined using a Dunnett post-hoc test.

We next investigated whether the isoproterenol-induced dephosphorylations are dependent on phosphatase activation by pretreating MEFs prior to stimulation with the phosphatase inhibitor okadaic acid, a potent inhibitor of protein phosphatase 1, 2A, and 2B. Pretreatment with okadaic acid did not prevent the isoproterenol-induced dephosphorylations of GSK3β(S9), PP1α(T320), p70S6K(T389), and AKT(T308) (Figure 4). In a similar fashion, okadaic acid also did not prevent the same proteins from being dephosphorylated when MEFs were stimulated with forskolin (data not shown). Nonetheless, we found that okadaic acid increased the basal level of phosphorylation of p70S6K(T389), GSK3β(S9), and PKA substrate, suggesting that an okadaic acid-sensitive phosphatase is involved in maintaining the basal level of phosphorylation of these proteins. We further observed that okadaic acid blocked the initial rise and the secondary decline in the phosphorylation of PKA substrate after isoproterenol stimulation. However, this was likely secondary to the higher basal levels of protein phosphorylation in the presence of okadaic acid.

**Figure 5:**
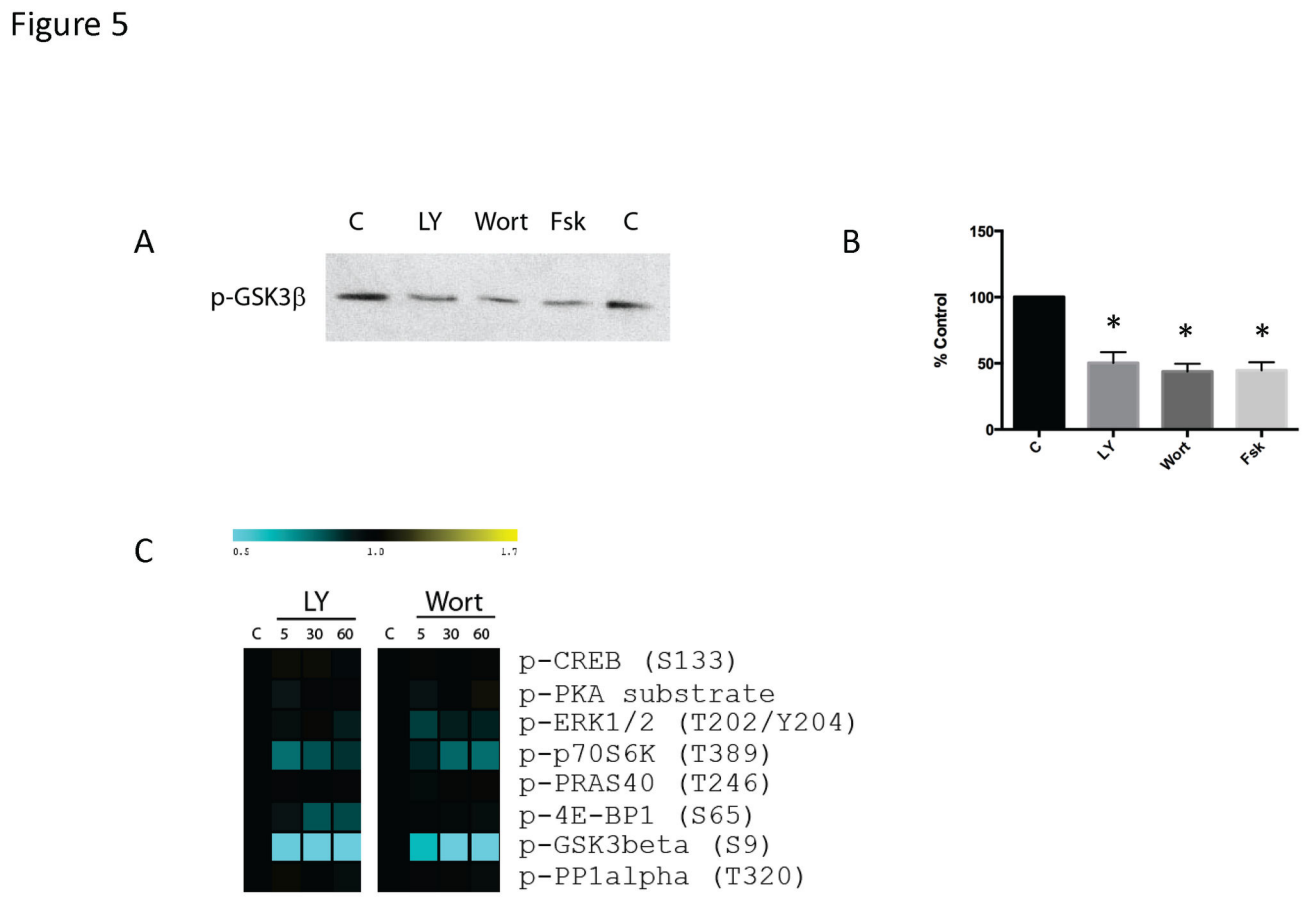
Inhibition of PI3K/AKT results in dephosphorylation of downstream signaling molecules. **A**, In vitro AKT kinase assay. MEFs were treated with vehicle control (C), LY294002 (LY) (10 μM), wortmannin (Wort) (100 nM) or forskolin (Fsk) (50 μM) for 30 minutes. MEF lysates were collected and were assayed for AKT activity using an in vitro kinase assay. As a readout for AKT activity, phosphorylated GSK3β fusion protein was detected by western blotting. A representative blot is shown. B, The quantification of the AKT kinase assay. The graph shows mean ± SEM values for three independent experiments. * indicates a significant difference (P<0.05) as compared with the control phosphorylation level of GSK3β fusion protein. **C**, Phosphoprotein signaling in the presence of PI3K inhibitors. MEFs were treated with LY294002 (LY) (10 μM) or wortmannin (Wort) (100 nM) for various times (0, 5, 30, and 60 minutes). The heat map shows phosphorylation (yellow) and dephosphorylation (blue) events after the various treatments. Data are representative of two independent experiments.

In reviewing the list of signaling molecules that exhibited isoproterenol- and forskolin- induced patterns of dephosphorylation, we consistently observed a significant dephosphorylation of AKT at threonine 308. Furthermore, we recognized that many of the other proteins that were subject to dephosphorylation are known targets of the PI3K/AKT signaling pathway (e.g., GSK3β, p70S6K, PRAS40, and 4E-BP1) [24]. This led us to hypothesize that cAMP, generated downstream of β-AR, inhibits AKT activation. First, we investigated the effects of forskolin on AKT kinase activity using an in vitro AKT kinase assay. As can be seen in Figure 5A and B, treatment with forskolin led to a decrease in the phosphorylation of the AKT substrate, GSK3β, indicating that AKT kinase activity was inhibited by forskolin through a cAMP-dependent mechanism. We also observed that AKT kinase activity was inhibited by treatment with LY294002 and wortmannin and that these PI3K inhibitors could induce dephosphorylations in certain proteins such as GSK3β(S9), P70S6K(T389), and 4E-BP1(S65) similar to as seen with forskolin (Figure 5C). 

Of note, we observed that one of these forskolin-induced dephosphorylation events PP1α(T320), could not be reproduced with treatment with LY294002 and wortmannin, suggesting that PP1α(T320) is regulated in an AKT-independent fashion. Interestingly, this dephosphorylation event, which is known to result in increased PP1 activity [25], coincided with the secondary decline in phosphorylation of various PKA substrates, indicating that these signaling events are linked. Together, these results are supportive of two links between cAMP and the protein dephosphorylations that we observed: 1) a link between cAMP and PI3K/AKT inhibition and 2) a link between cAMP and dephosphorylation of PP1α at threonine 320. 

## Discussion

Using the lysate microarray technique, we conducted broad-scale phosphoprotein profiling of signaling molecules downstream of β-AR activation. This technique facilitated the simultaneous screening of a large number of samples with many different antibodies and provided us with a comprehensive view of the signaling network downstream of β-AR activation that would have otherwise not been possible using traditional western blot analysis. Using this approach we identified a number of novel phosphorylation and dephosphorylation events induced by β-AR stimulation. These studies, thus, shed light on the complexity of β-AR signaling.

In addition to detecting phosphorylation events such as ERK1/2(T202/Y204) and CREB(S133), which are known to be downstream of PKA in β-AR signaling, our approach detected a number of novel tyrosine phosphorylation events in response to isoproterenol stimulation: Cdc2(Y15) and Pyk2(Y402). These phosphorylations were dependent on PKA as they were blocked by treatment with H89, however they occurred on a much slower timescale than ERK1/2(T202/Y204) and CREB(S133) suggesting that they were more distal in the pathway. The phosphorylation of Cdc2(Y15) downstream of PKA could have occurred either through activation of Wee1 kinase or deactivation of the Cdc2 effector phosphatase Cdc25 [26], as previous studies have linked PKA to the activity of these enzymes. Post-translational modification at tyrosine 15 on Cdc2 is known to be an inhibitory phosphorylation event on this protein and is expected to prevent cell entry into mitosis and, therefore, may be an important mechanism by which β-AR agonists regulate the cell cycle [27]. The second novel phosphorylation event that we observed was Pyk2(Y402), which is expected to increase Pyk2 activity in MEFs [28]. Pyk2 is a protein tyrosine kinase that is involved in cell spreading and migration [28], and may have a pro-apoptotic role in mouse fibroblasts [29]. Thus, detection of these novel phosphorylation events provides new insights into how β-AR activation may negatively impact the proliferation and survival of certain cells.

**Figure 6:**
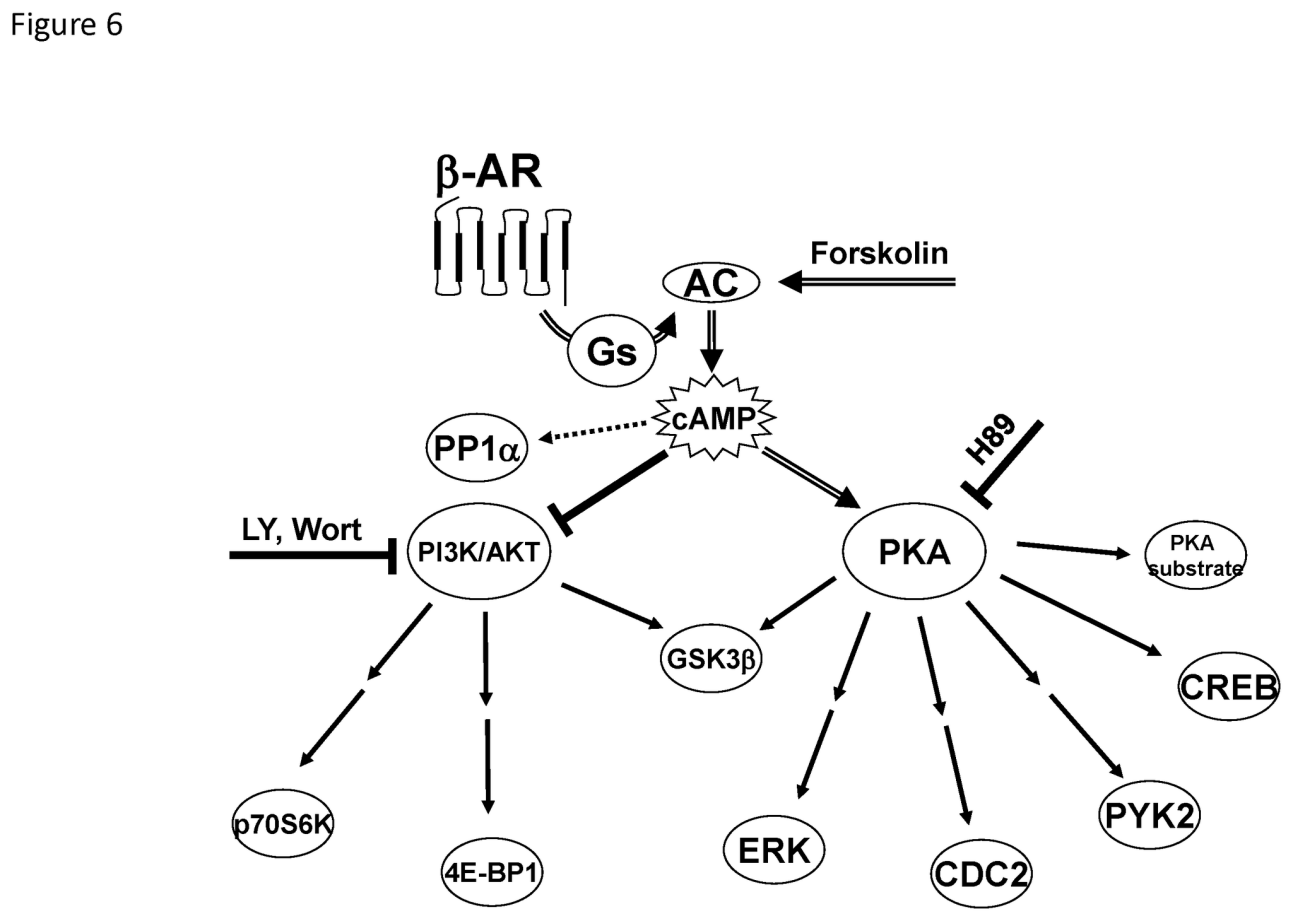
A model of phosphoprotein signaling mediated by β-AR agonists and forskolin. Cyclic AMP (cAMP) is generated by activation of adenylyl cyclase (AC) through β-AR stimulation or by direct activation by forskolin. PKA is then activated by cAMP and phosphorylates a variety of targets either directly (single arrow) or through intermediates (sequential arrows). Independent of PKA kinase activity, cAMP can inhibit basal PI3K/AKT activity leading to the dephosphorylation of downstream targets for PI3K/AKT. PP1α is also dephosphorylated by agents that increase cAMP levels, but this dephosphorylation is not dependent on PI3K/AKT inhibition, as neither treatment with LY294002 (LY) nor wortmannin (Wort) led to dephosphorylation of PP1α. GSK3β can be phosphorylated by both PKA and AKT. When MEFs are stimulated by isoproterenol or forskolin in the presence of H89 (PKA inhibitor), enhanced dephosphorylation of GSK3β is observed because the rapid phosphorylation of GSK3β by PKA is blocked. Basal phosphorylation of GSK3β by AKT is inhibited by cAMP leading to the dephosphorylation of GSK3β. Solid arrows represent phosphorylation, double line arrows represent activation, and blocked lines represent inhibition.

Another insight from these lysate microarray studies is that GSK3β(S9) can be phosphorylated by both PKA and AKT as shown in the model (Figure 6). GSK3β is a regulator of glycogen synthesis that is known to be inhibited by phosphorylation on serine 9 by AKT [30]. Our studies confirm this relationship since treatment of MEFs with PI3K inhibitors produced a dephosphorylation of GSK3β(S9). Prior studies have also demonstrated that PKA can directly phosphorylate GSK3β(S9) [31] and we observed rapid phosphorylation of GSK3β(S9) with either isoproterenol or forskolin that was blocked by the PKA inhibitor H89. GSK3β may thus be an important signaling node whose phosphorylation status reflects input from activation of both PKA and PI3K/AKT pathways. 

We observed that β-AR activation also led to a number of key dephosphorylation events in protein targets of PI3K/AKT: GSK3β(S9), 4E-BP1(S65), and p70S6K(T389). PI3K/AKT is a master regulator of cell growth, and activation of this signaling pathway is critical for cell survival and is upregulated in many cancers [32]. We demonstrated that forskolin inhibits AKT kinase activity in MEFs and that inhibitors of PI3-kinase could generate the same dephosphorylation events. The β-AR-induced dephosphorylation events were PKA-independent because they were not blocked by H89 pretreatment; they were also not reproduced with EPAC agonists. Our finding that isoproterenol stimulation reduced AKT activity is interesting and matches a previous report that showed a link between cAMP activation and reduction of PI3K/AKT pathway in human diffuse large B cell lymphoma cells [33]. Similar to our study, this report found that cAMP inhibited PI3K/AKT in the lymphoma cells by a mechanism that was independent of PKA and Epac [33]. Furthermore, inhibition of PI3K/AKT by cAMP has also been reported in COS cells, a fibroblast-like cell line [34]. In this report, the authors found that cAMP inhibited AKT by blocking the membrane association of AKT with its upstream regulator PDK1. Similarly, a study of dermal fibroblasts showed that the growth of these cells was inhibited by β2-AR activation and that this was associated with a dephosphorylation of AKT [35]. However, these results are in contrast with other studies that have shown that β2-AR can positively couple to AKT. For example, β2-AR has been shown to activate PI3K/AKT via a Gi-dependent process in cardiac myocytes [7]. We speculate that this differential coupling to PI3K/AKT may be a mechanism to preserve some tissues while inhibiting growth of other tissues during stressful periods when catecholamine levels are high.

It is still unclear how cAMP inhibited PI3kinase/AKT in our MEF cells. One possibility is that it occurs through another unknown cAMP sensor. It is also possible that this effect on PI3kinase/AKT occurred through the regulatory units of PKA. Although H89 inhibits the catalytic activity of PKA, it does not prevent the dissociation of PKA into its catalytic and regulatory units, and a prior study showed that the regulatory units of PKA may have important signaling functions [36]. Future studies are needed to understand how β-AR agonists signaling through cAMP can regulate the activity of PI3K/AKT.

The other dephosphorylation event that was cAMP-dependent, but independent of PI3K was of PP1α(T320). PP1α is a central regulator of protein phosphorylation that also has been shown to control cell cycle progression [37]. Dephosphorylation of PP1α at threonine 320 is known to result in activation of this phosphatase [25] and this activity has been linked to both G1 arrest of human cancer cells [38] and promotion of apoptosis in HL-60 cells concurrent with dephosphorylation of the retinoblastoma protein pRB [39]. Thus, cAMP-dependent activation of PP1α may be another way by which β-AR agonists may have anti-proliferative and pro-apoptotic effects. 

Our data also suggest that activation of PP1α may be part of a feedback loop by which cAMP returns PKA-phosphorylated substrates to basal state. In support of this concept is the finding that dephosphorylation of PP1α(T320) was a delayed β-AR signaling event that coincided with the secondary decline in phosphorylation levels of PKA substrate and CREB post stimulation. Although not specific for PP1, okadaic acid also increased the basal phosphorylation levels of PKA substrate, resulting in no induction and no secondary decline in phospho-substrate after β-AR stimulation. In the heart, PP1 has been shown to dephosphorylate protein targets that are phosphorylated by PKA, such phospholambam [40]. PP1 activity is also enhanced in heart failure and may be a part of homeostatic mechanism to counterbalance chronic β-AR activation [41]. In addition to PP1, previous literature showed that cAMP could activate another phosphatase, PP2, in a PKA independent fashion in NRK-52E cells [42], suggesting that there may be a broader activation of phosphatases by cAMP. Further work is necessary to determine the mechanism of how cAMP promotes PP1α(T320) dephosphorylation and to explore the hypothesis that this is a way of extinguishing β-AR signaling in MEFs and other cell types. 

In summary, we propose the following model of β-AR signaling to explain the observed phosphorylation and dephosphorylation events (Figure 6). The phosphorylation events (e.g., ERK1/2(T202/Y204), CREB(S133), Cdc2(Y15), Pyk2(Y402), and PKA substrate that we observed downstream of β-AR activation were all dependent on cAMP and PKA because they were reproduced by forskolin and were blocked by treatment with PKA inhibitor H89. Based on our studies with the selective β-AR antagonists, we also observed that β2-ARs have a greater capacity than β1-ARs to couple to these phosphorylation events. We reason that this may be due to the regulation of distinct pools of cAMP by the different β-AR subtypes as has been observed in other cells [22,43]. This may also be related to the finding that β2-AR are the major β-AR subtype in MEFs. On the other hand, most of the dephosphorylation events that we observed (e.g., GSK3β(S9), 4E-BP1(S65), p70S6K(T389)) were independent of PKA activity and, instead, were mediated by cAMP inhibition of PI3K/AKT (Figure 6), with the exception of GSK3β which was targeted by both pathways. Both β-AR subtypes coupled equally well to the dephosphorylation events, suggesting that they are regulated by a common pool of cAMP. In addition, we identified an example of a β-AR-induced dephosphorylation event, PP1α(T320), that appeared to be independent of the decrease in PI3K/AKT and may play a role in extinguishing the phosphorylation of certain PKA substrates to terminate signaling. 

In conclusion, we have shown how application of lysate microarrays can be used to provide a greater understanding of phosphoprotein signaling for a specific pathway. By applying lysate microarrays to β-AR signaling in MEFs, we identified novel β-AR signaling events, including protein phosphorylations and dephosphorylations. Further delineation of these pathways in MEFs and other cell types will be important for defining a role for β-AR agonists/antagonists in cancer treatment and will be useful to understanding how states of chronic catecholamine excess (e.g., heart failure) can lead to proliferation or apoptosis of different cell types. 

## Supporting Information

File S1 **Figures S1-S6.** Figure S1. Competition binding study in MEFs with ICI 118,551. Membranes were prepared from MEFs and were used in binding reactions. For each reaction, 60 μg of membranes was incubated with 10 nM [^3^H]dihydroalprenolol ([^3^H]DHA) and varying concentrations of the β2-AR selective antagonist ICI 188,551. Non-specific binding was determined in the presence of 1 μM alprenolol. Results show mean ± SEM values from three experiments. Competition data were best fit with a two-site model that included a high-affinity binding site (74.7%) and low-affinity binding site (25.3%) for ICI 118,551. The high-affinity site corresponds to β2-AR, whereas low-affinity sites are β1-AR or β3-AR. Figure S2. Phosphoprotein signaling in MEFs after stimulation with epinephrine. A heat map representation of phosphoprotein changes over time. MEFs were grown to confluence and then were stimulated with different doses of epinephrine (Epi) (1 μM, 10 μM, or 100 μM) for various times (0, 5, 10, 20, 30, or 60 minutes). Stimulations were performed in the presence or absence of the alpha 1 adrenergic receptor antagonist prazosin (Praz) (1 μM). Using data from the lysate microarrays, a heat map was constructed which revealed distinct clusters of phosphorylation (yellow) and dephosphorylation (blue) events after epinephrine stimulation. The color scale shows fold change as compared with unstimulated MEFs. Data are representative of two independent experiments. Figure S3. Phosphoprotein signaling in MEFs after stimulation with norepinephrine. A heat map representation of phosphoprotein changes over time. MEFs were grown to confluence and then were stimulated with different doses of norepinephrine (Norepi) (1 μM, 10 μM, or 100 μM) for various times (0, 5, 10, 20, 30, or 60 minutes). Stimulations were performed in the presence or absence of the alpha 1 adrenergic receptor antagonist prazosin (Praz) (1 μM). Using data from the lysate microarrays, a heat map was constructed which revealed distinct clusters of phosphorylation (yellow) and dephosphorylation (blue) events after norepinephrine stimulation. The color scale shows fold change as compared with unstimulated MEFs. Data are representative of two independent experiments. Figure S4. Western blotting confirmatory studies on MEF lysates used for lysate microarrays. Figures show phospho (denoted by “p-“) and total levels of proteins as detected by western blotting. Each blot represents an isoproterenol time course study with 0, 5, 10, 20, 30 and 60 minute stimulations. Dephosphorylated proteins are shown at the top of the figure and phosphorylated proteins are shown at the bottom of the figure. Figure S5. Isoproterenol induced phosphoprotein signaling in the presence of pertussis toxin. MEFs were pretreated with pertussis toxin (PTX) (1 μg/ml) or vehicle control for 30 minutes and then stimulated with isoproterenol (Iso) (1 μM) for various times (0, 5, 10, 20, 30, or 60 minutes). A heat map shows phosphorylation (yellow) and dephosphorylation (blue) events after isoproterenol stimulation. The isoproterenol time course study on the left was performed in the presence of PTX and the time course study on the right was performed in the presence of vehicle. The color scale shows fold change as compared with unstimulated MEFs. Data are means of three independent stimulation experiments. Figure S6. Epac activation as assessed by the Rap GAP assay. MEFs were stimulated with vehicle control (C), forskolin (FSK) (50 μM), or the Epac-specific analog 8-pCPT-2'-O-Me-cAMP (Epac) (250 μM) for 5 or 20 minutes. Following stimulation, cells were lysed and incubated with Ral GDS-Rap Binding Domain bound to glutathione-agarose beads to specifically pull down Rap1-GTP (see Materials and Methods). Western blotting was then performed to detect Rap1. Positive (+) and negative (-) controls are included in the figure. Data depicted are representative of two independent stimulation experiments.
(PDF)
