# Supplementary figures and images for: Broad-Scale Phosphoprotein Profiling of Beta Adrenergic Receptor (β-AR) Signaling Reveals Novel Phosphorylation and Dephosphorylation Events
Source: PLoS One. 2013 Dec 5;8(12):e82164. doi: 10.1371/journal.pone.0082164 (PMC3855414; doi:10.1371/journal.pone.0082164)

Figure S1

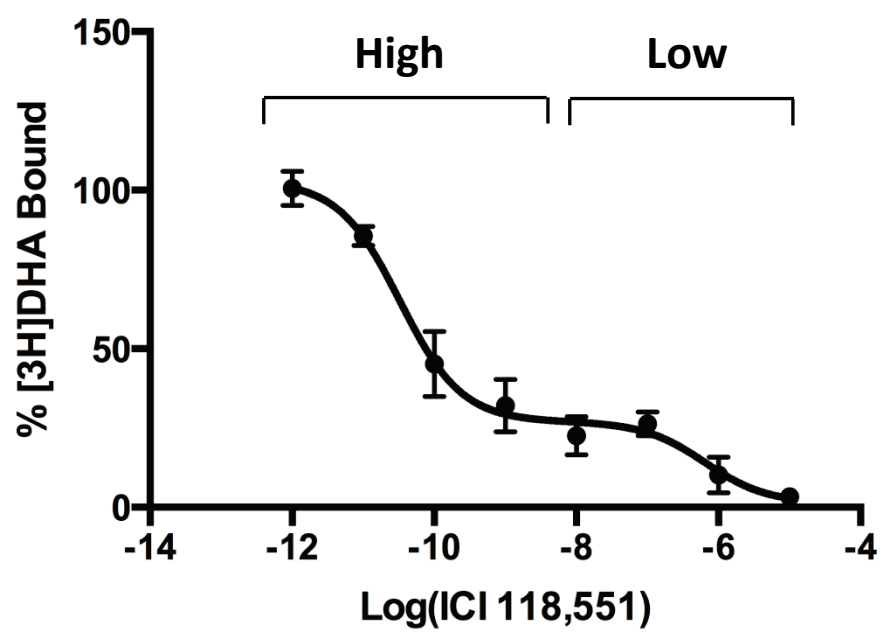

Figure S2

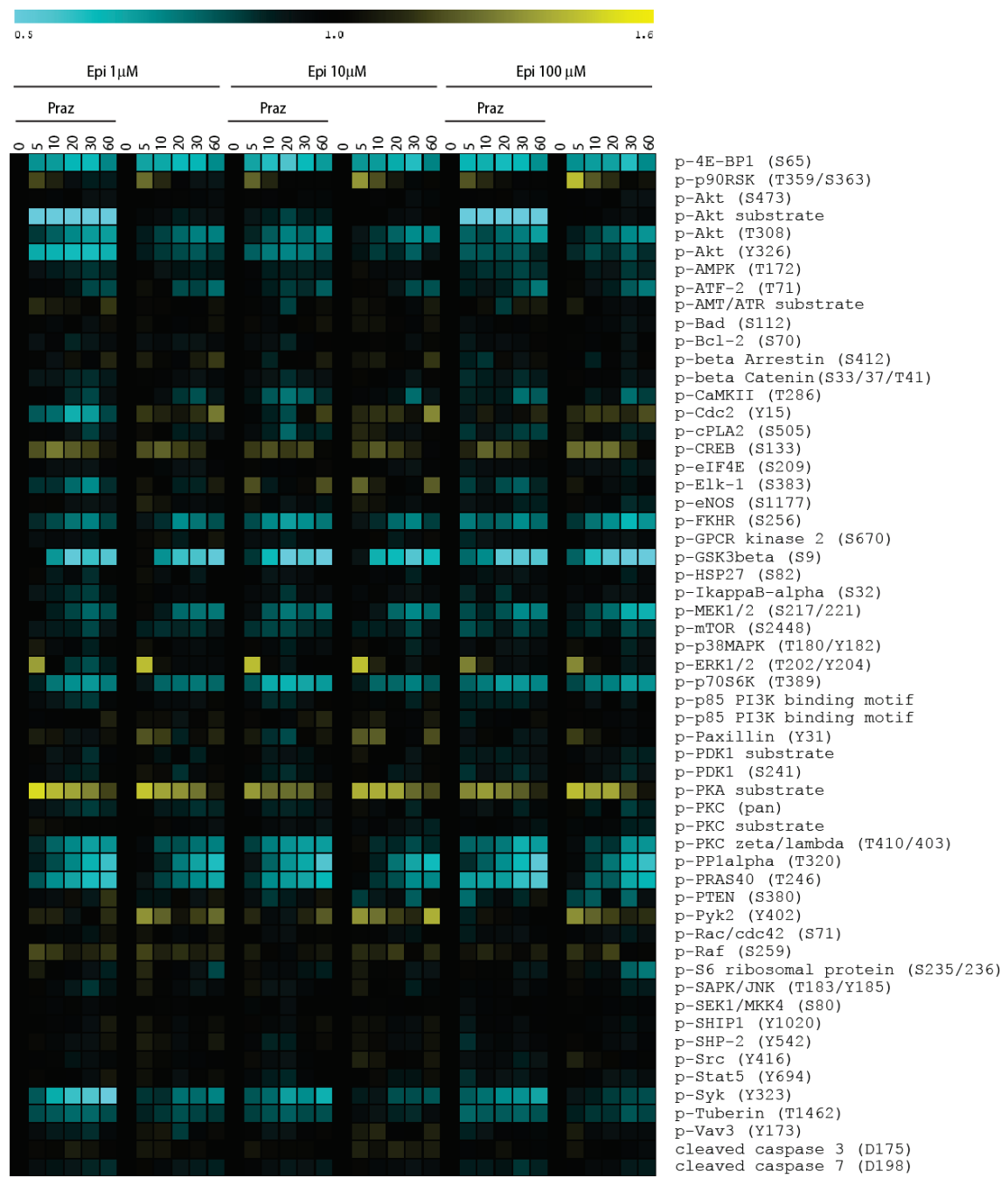

Figure S3

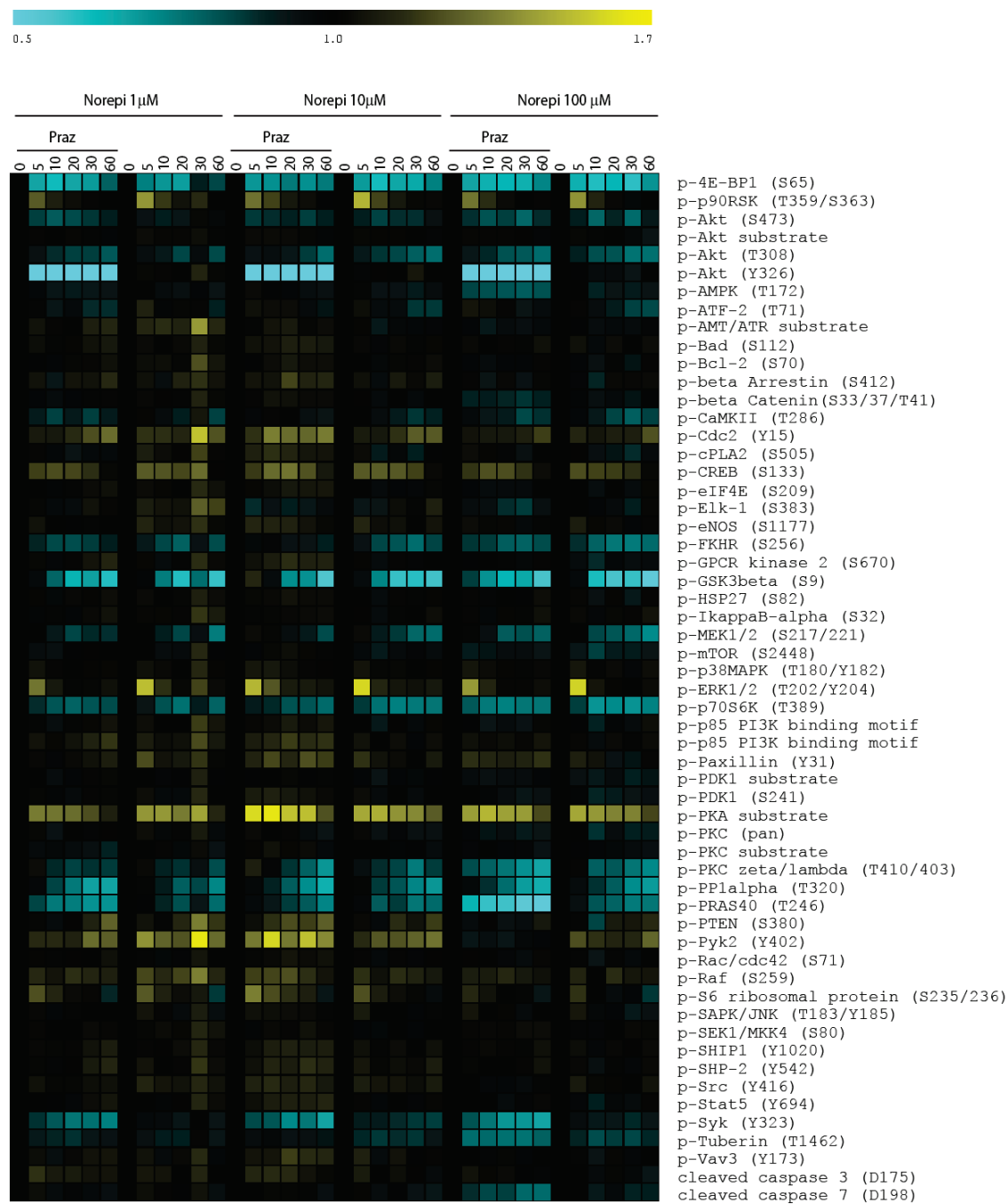

Figure S4

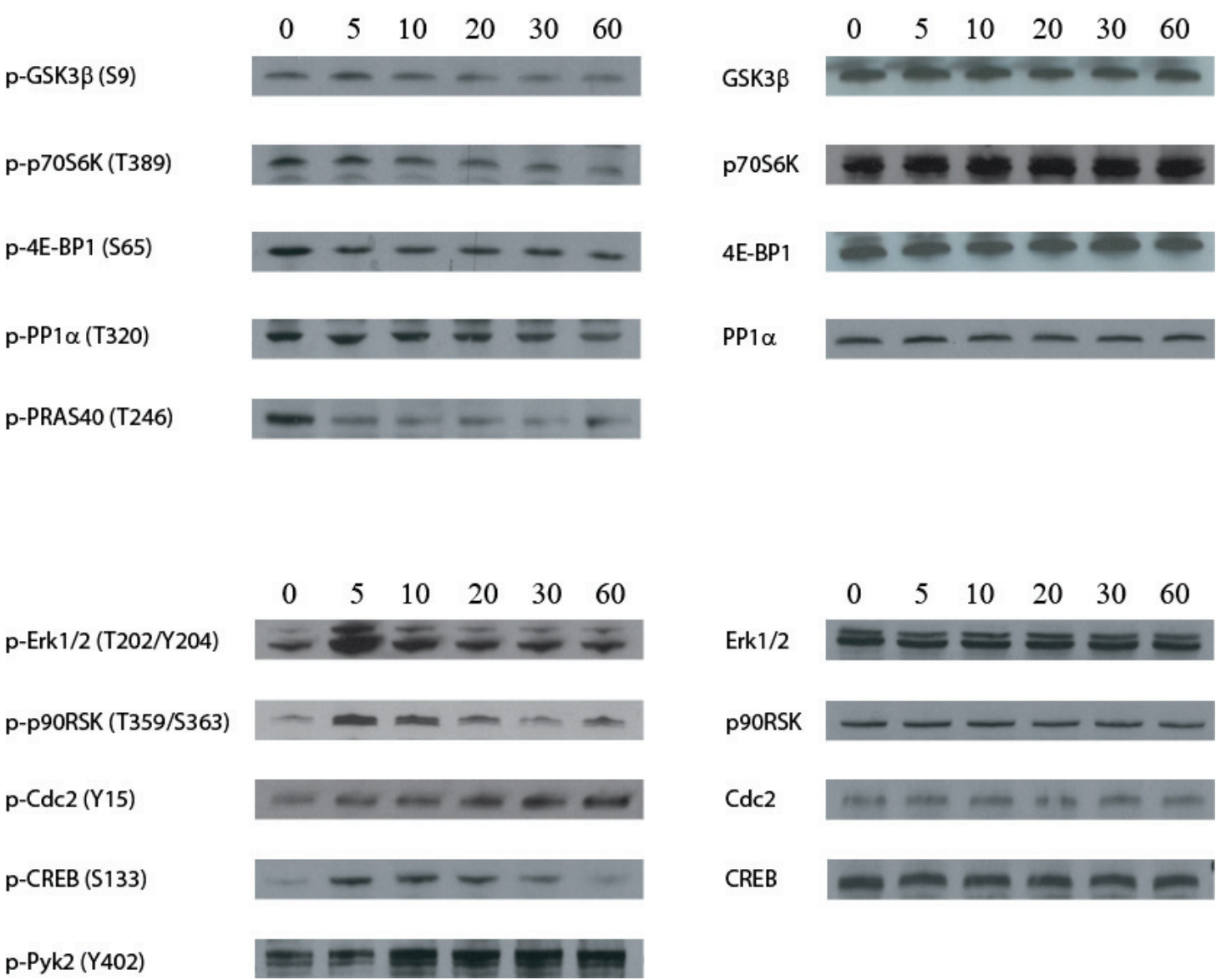

Figure S5

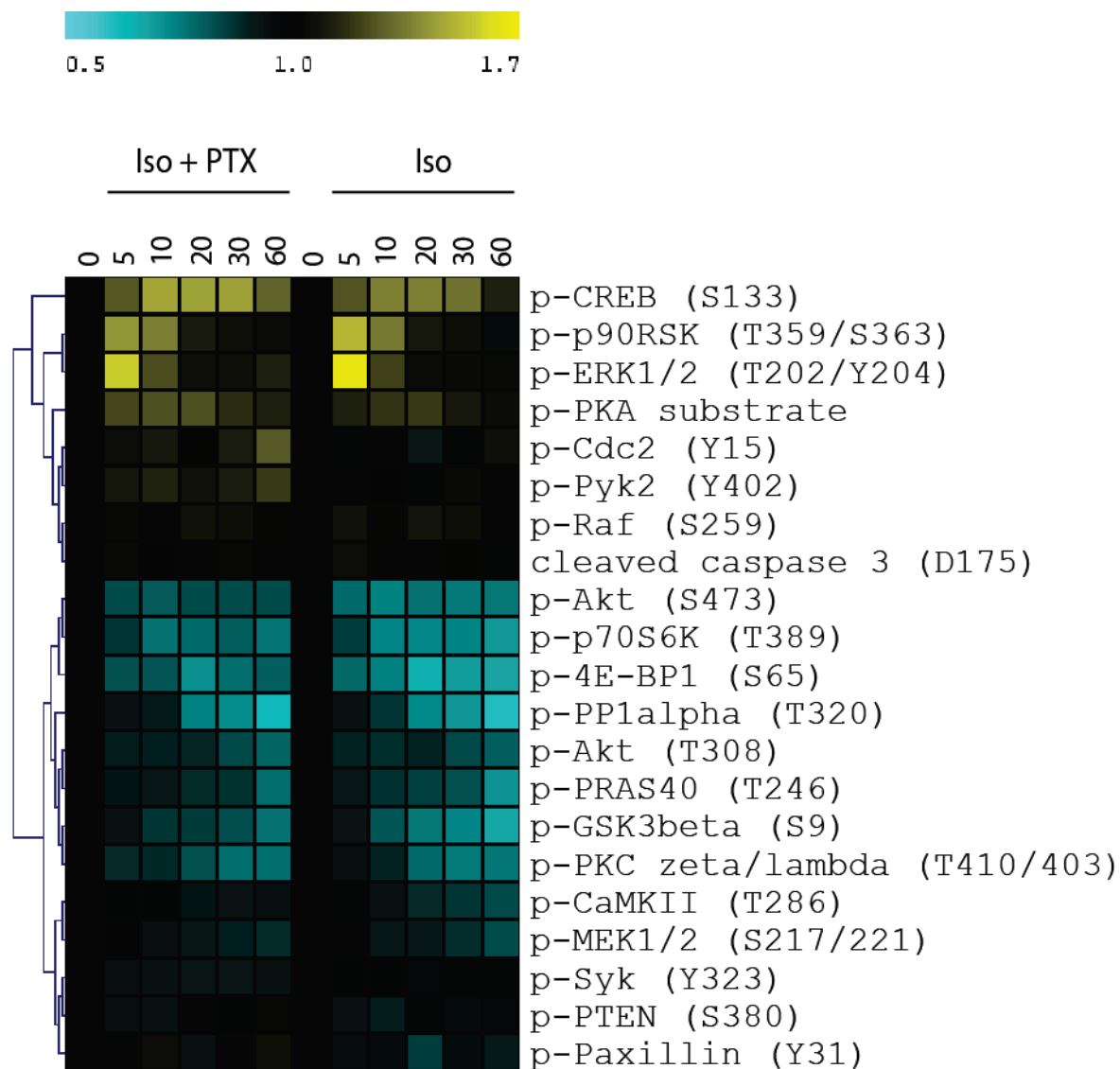

Figure S6

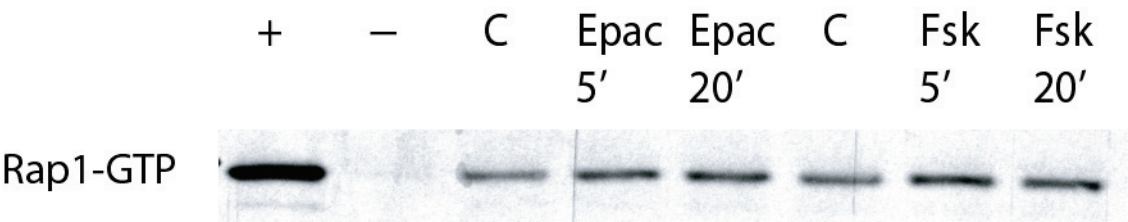

Supplement: File S1 — Figures S1-S6. Figure S1. Competition binding study in MEFs with ICI 118,551. Membranes were prepared from MEFs and were used in binding reactions. For each reaction, 60 μg of membranes was incubated with 10 nM [3H]dihydroalprenolol ([3H]DHA) and varying concentrations of the β2-AR selective antagonist ICI 188,551. Non-specific binding was determined in the presence of 1 μM alprenolol. Results show mean ± SEM values from three experiments. Competition data were best fit with a two-site model that included a high-affinity binding site (74.7%) and low-affinity binding site (25.3%) for ICI 118,551. The high-affinity site corresponds to β2-AR, whereas low-affinity sites are β1-AR or β3-AR. Figure S2. Phosphoprotein signaling in MEFs after stimulation with epinephrine. A heat map representation of phosphoprotein changes over time. MEFs were grown to confluence and then were stimulated with different doses of epinephrine (Epi) (1 μM, 10 μM, or 100 μM) for various times (0, 5, 10, 20, 30, or 60 minutes). Stimulations were performed in the presence or absence of the alpha 1 adrenergic receptor antagonist prazosin (Praz) (1 μM). Using data from the lysate microarrays, a heat map was constructed which revealed distinct clusters of phosphorylation (yellow) and dephosphorylation (blue) events after epinephrine stimulation. The color scale shows fold change as compared with unstimulated MEFs. Data are representative of two independent experiments. Figure S3. Phosphoprotein signaling in MEFs after stimulation with norepinephrine. A heat map representation of phosphoprotein changes over time. MEFs were grown to confluence and then were stimulated with different doses of norepinephrine (Norepi) (1 μM, 10 μM, or 100 μM) for various times (0, 5, 10, 20, 30, or 60 minutes). Stimulations were performed in the presence or absence of the alpha 1 adrenergic receptor antagonist prazosin (Praz) (1 μM). Using data from the lysate microarrays, a heat map was constructed which revealed distinc [file pone.0082164.s001.pdf]
